# Predictors of a prolonged puncture-wire time in patients with ST-Elevation Myocardial Infarction (STEMI)

**DOI:** 10.1186/s12872-026-06220-x

**Published:** 2026-07-07

**Authors:** Merve Günes-Altan, Stephan Achenbach, Johannes Michael Altstidl, Maximilian Moshage, Henry Strupp, Lennart Lorenz, Stephan Scholl, Mohamed Marwan, Monique Troebs, Luise Gaede

**Affiliations:** 1 Department of Cardiology and Angiology, University Hospital Erlangen, Friedrich-Alexander University Erlangen-Nürnberg (FAU), Ulmenweg 18, Erlangen, 91054 Germany; 2 Department of Cardiology, Klinikum Herrsching, Starnberger Kliniken, Herrsching, Germany

**Keywords:** ST-elevation myocardial infarction (STEMI), Percutaneous coronary intervention (PCI), Puncture-to-wire time, Reperfusion delay

## Abstract

**Background:**

In patients with ST-elevation myocardial infarction (STEMI), rapid reperfusion is essential for optimal outcomes, yet factors influencing intraprocedural delays remain insufficiently investigated.

**Methods:**

In this single-center retrospective study, consecutive STEMI patients undergoing primary percutaneous coronary intervention (PCI) between 2015 and 2024 were analyzed. Prolonged time between vascular puncture and wire passage (puncture-wire time; PWT) was defined as a value in the fourth quartile of the study population (>17.1 minutes). Multivariable logistic regression identified independent predictors and procedural determinants of prolonged PWT.

**Results:**

A total of 1,235 patients (70.7% male, median age 64.5 years [IQR 56.0–75.25]) were included. Radial-to-femoral access crossover (OR 2.539, 95% CI 1.381–4.665, p = 0.003), arterial kinking or severe radial spasm (OR 3.669, 95% CI 1.986–6.778, p < 0.001) and challenging revascularization of the culprit lesion (OR 2.330, 95% CI 1.319–4.116, p = 0.004) were independently associated with prolonged PWT. Direct intervention of the culprit lesion was independently associated with a lower likelihood of prolonged PWT (OR 0.445, 95% CI 0.201 – 0.988, p = 0.047). In contrast, the primary choice of vascular access (radial vs. femoral) was not associated with prolonged PWT (p=0.06).

**Conclusion:**

While some factors are system- or patient-dependent, early recognition of procedural difficulties and individualized access-site decision making may help minimize procedural delays. In selected patients, a primary femoral access route may be considered, and direct intervention of the culprit lesion should be pursued to minimize PWT.

**Clinical trial number:**

Not applicable.

**Supplementary Information:**

The online version contains supplementary material available at 10.1186/s12872-026-06220-x.

## Introduction

ST-elevation myocardial infarction (STEMI) requires rapid reperfusion to limit myocardial damage and improve clinical outcomes [1–3]. Numerous studies have consistently demonstrated a strong association between shorter reperfusion times and reduced mortality, underscoring that every minute of delay adversely affects prognosis [3, 4]. Consequently, current guidelines recommend strict time targets for reperfusion in patients undergoing primary percutaneous coronary intervention (PCI) [5]. 

To better characterize sources of treatment delay, breaking down composite reperfusion metrics such as the door-to-balloon time into smaller procedural intervals may provide granular insight into potentially modifiable contributors of reperfusion delay. One such interval is the time between arterial puncture and wire crossing (puncture-to-wire time, PWT), which may help may help identify procedure-related delays and potential targets for optimization.

Despite its potential clinical relevance, determinants of prolonged PWT have not been systematically investigated. A better understanding of factors influencing PWT may identify opportunities to optimize procedural strategies, particularly with regard to vascular access selection.

Therefore, the aim of the present study was to identify predictors and procedural determinants of prolonged PWT and their clinical implications, providing insight into procedural strategies that may improve efficiency and patient outcomes.

## Methods

### Study design

This single-center, real-world registry analysis includes 1,403 patients presenting with STEMI undergoing coronary angiography from January 2015 to December 2024 in the department of cardiology and angiology, University Hospital Erlangen-Nuremberg. Patients were included if they met contemporary diagnostic criteria for STEMI: symptoms compatible with acute myocardial ischemia together with persistent ST-segment elevation on a 12-lead ECG in at least two contiguous leads, using the European Society of Cardiology (ESC) guideline thresholds [6]. Of these, 1235 patients underwent primary PCI and constituted the final study population. Patients not undergoing PCI represented a heterogeneous group, including patients without an angiographically identifiable culprit lesion, spontaneous reperfusion, alternative diagnoses despite initial STEMI activation, referral for urgent surgical revascularization, inability to cross the culprit lesion with a guidewire, or advanced native coronary or graft disease not amenable to PCI.

### Assessments

Baseline demographic and clinical characteristics including age, cardiovascular risk factors and medical history were recorded. Puncture time was defined as the first injection of local anesthesia at the intended puncture site, wire passage was defined as the moment when the wire crossed the culprit lesion. Primary access site was recorded as the first attempted access site. Core working hours were defined as weekdays between 07:00 a.m. and 06:00 p.m. Procedures performed outside core working hours were defined as interventions initiated outside regular daytime working hours, including night-time, weekends, and public holidays. Challenging coronary access was defined as the use of more than one catheter to intubate the targeted coronary artery, any atypical origin of a coronary artery, the presence of an ostial stent, an aneurysm of the ascending aorta as well as any coronary artery anomaly including separate origins of LAD/RCX. Challenging revascularization of the culprit lesion was defined as the need of more than one wire to achieve passage, the use of a dedicated chronic total occlusion (CTO) wire as well as microcatheter-assisted or balloon-assisted techniques. Direct intervention of the culprit lesion was defined as direct engagement of the anticipated culprit vessel with a guiding catheter and initiation of PCI before completion of diagnostic angiography of the remaining coronary arteries.Culprit lesion localization was classified as left anterior descending artery (LAD), left circumflex artery (LCX), or right coronary artery (RCA). Lesions involving the left main coronary artery or other atypical localizations were excluded from this analysis due to low event numbers. First medical contact (FMC)-to-door time was defined as the interval between first contact with emergency medical services and hospital arrival.

Door-to-puncture time was defined as the interval between hospital arrival and first injection of local anesthesia at the intended vascular access site. FMC and hospital arrival times were obtained from emergency medical service (EMS) records.

Puncture-to-wire time (PWT) was defined as the interval between first injection of local anesthesia at the puncture site and guidewire crossing of the culprit lesion. Puncture time was documented in the catheterization laboratory report, and wire crossing time was determined from angiographic imaging timestamps.

All time intervals were recorded in minutes.

**Fig. 1:**
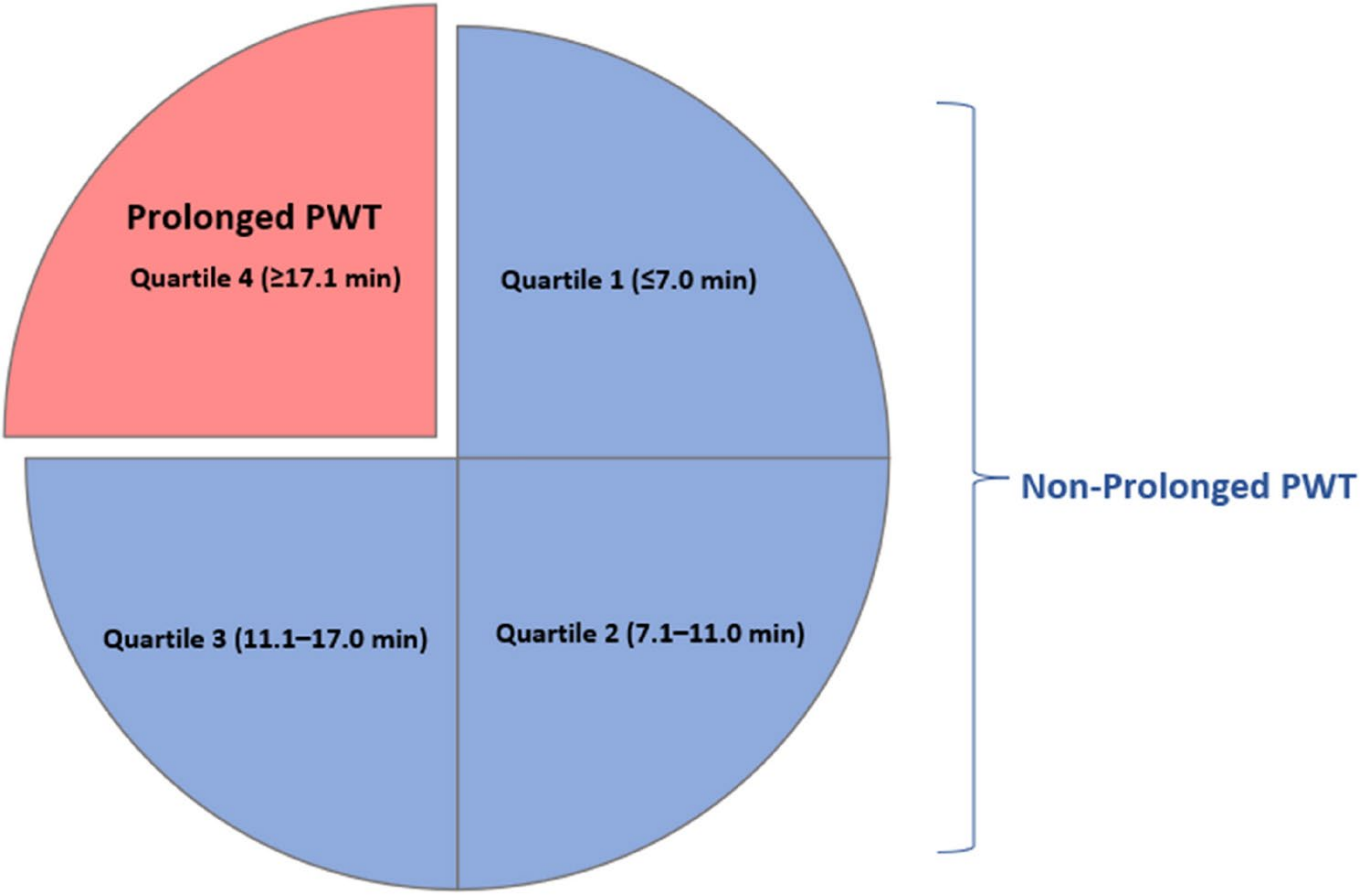
Quartiles based on puncture-wire time (PWT)

Patients were categorized into quartiles according to their PWT, based on the overall distribution within the study cohort: quartile 1 (≤ 7 min), quartile 2 (7.1–11 min), quartile 3 (11.1–17 min) and quartile 4 (≥ 17.1 min) (Fig. 1). Patients in Quartile 4 were defined as patients with prolonged PWT, whereas quartiles 1–3 were considered to reflect non-prolonged PWT. To identify predictors of a prolonged PWT, baseline clinical and procedural characteristics were compared patients with or without prolonged PWT. An additional analysis compared clinical outcomes between patients with or without prolonged PWT. Adverse in-hospital outcome was defined as the composite of in-hospital death, acute kidney injury (AKI) and left ventricular ejection fraction (LVEF) < 30%. Acute kidney injury was defined according to the Acute Kidney Injury Network (AKIN) criteria, based on an increase in serum creatinine within 48 h after revascularization. Composite endpoints were selected to capture clinically relevant adverse in-hospital consequences potentially associated with delayed reperfusion, including mortality, severe left ventricular dysfunction, and acute kidney injury. In addition, a procedural adverse outcome, defined as the composite of in-hospital death and post-procedural TIMI (Thrombolysis in Myocardial Infarction) flow < 3, was analysed.

### Statistical analysis

Statistical analysis was performed using SPSS Statistics 28.0 (IBM, Armonk, New York, USA). Continuous variables are expressed as mean ± standard deviation (SD) or median (interquartile range [IQR]), as appropriate, in text and tables. Categorical variables are expressed as numbers and percentages and were compared using the Chi-square test. Due to their skewed distribution, time variables were reported as median (IQR) and compared using the Mann–Whitney U test. Other continuous variables were compared using the unpaired Student’s t-test. Multivariable logistic regression analysis was performed to identify independent predictors of prolonged PWT. Multivariable logistic regression analysis was performed to identify independent predictors of prolonged PWT. Variables entered into the multivariable model were selected based on a predefined threshold of *p* < 0.10 in in univariable analyses and included patient-related factors (age, previous coronary artery bypass grafting (CABG), hemoglobin level at admission, presence of arterial hypertension, presence of diabetes mellitus, cardiogenic shock, and cardiopulmonary resuscitation (CPR) during the procedure), system-related factors (procedures performed outside core working hours, FMC-to-door time, door-to-puncture time), and procedural characteristics (primary access route, change of access route, arterial kinking or severe radial spasm, direct intervention of culprit lesion and challenging revascularization of the culprit lesion). Additional multivariable logistic regression analyses were performed for both composite endpoints to assess the independent association between prolonged PWT and clinical outcomes. Models were adjusted for baseline variables differing between groups (age, arterial hypertension, diabetes mellitus, and previous CABG). Odds ratios (OR) with 95% confidence intervals (CI) were calculated. A two-sided p-value < 0.05 was considered statistically significant.

## Results

### Clinical and procedural characteristics

A total of 1,235 patients were included in the analysis. Prolonged PWT was observed in 298 patients, whereas the remaining 937 patients were assigned to non-prolonged PWT. Baseline characteristics are summarized in Table 1, while clinical and procedural characteristics are detailed in Table 2.

**Table 1:** Baseline characteristics

	Non-Prolonged PWT (*n* = 937)	Prolonged PWT (*n* = 298)	*p*-value
Age at angiography (years)	64.6 ± 12.9	66.8 ± 14.2	0.015
BMI (kg/m²)	27.8 ± 7.6	27.5 ± 4.8	0.561
Heart rate (bpm)	80.9 ± 18.8	80.3 ± 17.9	0.615
Systolic BP (mmHg)	123.4 ± 26.1	124.4 ± 25.5	0.587
Male (n, %)	671 (71.6)	210 (70.5)	0.704
Comorbidities			
Arterial hypertension (n, %)	613 (66.2)	209 (72.8)	**0.036**
Diabetes mellitus (n, %)	216 (23.4)	84 (29.2)	0.051
Smoking (n, %)	465 (50.4)	132 (46.5)	0.323
Positive family history (n, %)	200 (21.7)	54 (19.0)	0.360
Previous PCI (n, %)	151 (16.2)	56 (19.2)	0.245
Previous CABG (n, %)	20 (2.2)	18 (6.2)	**0.001**
History of atrial fibrillation (n, %)	56 (6.0)	25 (8.7)	0.135

*BMI* Body Mass Index, *BP* Blood Pressure, *PCI *Percutaneous Coronary Intervention, *CABG* Coronary Artery Bypass Grafting

Bold values indicate statistically significant differences (*p* < 0.05)

**Table 2:** Clinical and procedural characteristics

	Non-Prolonged PWT (*n* = 937)	Prolonged PWT (*n* = 298)	*p*-value
Laboratory values			
Hemoglobin (g/dl)	13.8 ± 1.9	13.5 ± 2.3	**0.025**
CK at admission (U/I)	790.5 ± 1366.0	689.2 ± 1095.1	0.249
Creatinine at admission (mg/dl)	1.06 ± 0.6	1.09 ± 0.61	0.594
Time intervalls and specification			
Time pain-to- FMC (min)	89.0 [34.0–284.0]	104.50 [32.50–446.50]	0.107
FMC-to-door time (min)	44.0 [31.0–59.50]	48.50 [35.0–71.75]	**0.004**
Door-to-puncture time (min)	16.0 [9.0–34.0]	24.0 [11.0–54.0]	**< 0.001**
Procedure outside core working time (n, %)	498 (53.2)	195 (65.4)	**< 0.001**
Clinical presentation			
CPR before or during angiography (n, %)	136 (14.5)	59 (19.8)	**0.036**
CPR during angiography (n, %)	50 (5.3)	28 (9.4)	**0.019**
Cardiogenic shock (n, %)	233 (25.2)	109 (36.8)	**< 0.001**
Procedural characteristics			
Primary access route radial (n, %)	623 (66.5)	180 (60.4)	**0.06**
Change of primary access route from radial to femoral (n, %)	32 (3.4)	37 (12.4)	**< 0.001**
Arterial kinking / severe radial spasm (n, %)	28 (3.0)	38 (12.8)	**< 0.001**
Challenging revascularization of culprit lesion (n, %)	19 (2.0)	22 (7.4)	**< 0.001**
Direct intervention of culprit lesion (n, %)	78 (8.3)	9 (3.0)	**0.001**
Culprit lesion			
LAD (n, %)	435 (47.9)	130 (45.8)	
LCX (n, %)	112 (12.3)	48 (16.9)	
RCA (n, %)	362 (39.8)	106 (37.3)	0.140*

*CK* creatinine kinase, *FMC* First medical contact, *CPR * cardiopulmonary resuscitation, *LAD *left anterior descending artery, *LCX* left circumflex artery, *RCA* right coronary artery

Continuous variables are presented as mean ± standard deviation. Time variables are presented as median [interquartile range] and were compared using the Mann–Whitney U test

*p-value from Chi-square test comparing overall distribution

Bold values indicate statistically significant differences (*p* < 0.05)

Patients with prolonged PWT were significantly older (66.8 ± 14.2 vs. 64.6 ± 12.9 years, *p* = 0.015). There was a higher prevalence of arterial hypertension (72.8% vs. 66.2%, *p* = 0.036) and history of previous CABG was more prevalent among patients with prolonged PWT (6.2% vs. 2.2%, *p* = 0.001).

With regard to patient presentation, it was observed that prolonged PWT was more frequently observed outside core working hours (46.8% vs. 34.6%, *p* < 0.001). Cardiopulmonary resuscitation (CPR) prior to or during angiography was more frequent in patients with prolonged PWT (19.8% vs. 14.5%, *p* = 0.036), as was CPR during angiography (9.4% vs. 5.3%, *p* = 0.019). Cardiogenic shock at presentation was also more frequent in patients with prolonged PWT (36.8% vs. 25.2%, *p* < 0.001).

The time intervals prior to angiography also differed significantly. Patients with prolonged PWT experienced longer FMC-to-door times (80.1 ± 208.4 vs. 53.8 ± 64.7 min, *p* = 0.001) and longer door-to-puncture times (79.3 ± 398.6 vs. 30.5 ± 53.4 min, *p* < 0.001). There was no significant difference in pain-to-FMC time.

With regard to procedural factors, there was no significant difference between groups in terms of primary vascular access (primary radial access 60.4% vs. 66.5%, *p* = 0.06). However, patients in the group with prolonged PWT more frequently required a switch from radial to femoral access (12.4% vs. 3.4%, *p* < 0.001), and technical challenges appeared more often, such as arterial kinking or vascular spasm (12.8% vs. 3.0%, *p* < 0.001). In patients with prolonged PWT, the culprit lesion was less frequently treated directly (3.0% vs. 8.3%, *p* = 0.001) without completing the angiography first. With regard to culprit lesion localization, the distribution of infarct-related arteries did not differ between patients with prolonged and non-prolonged PWT (Table 2).

Laboratory values at admission showed lower hemoglobin levels in quartile 4 (13.5 ± 2.3 vs. 13.8 ± 1.9 g/dL, p = 0.025).

### Clinical endpoints

**Fig. 2:**
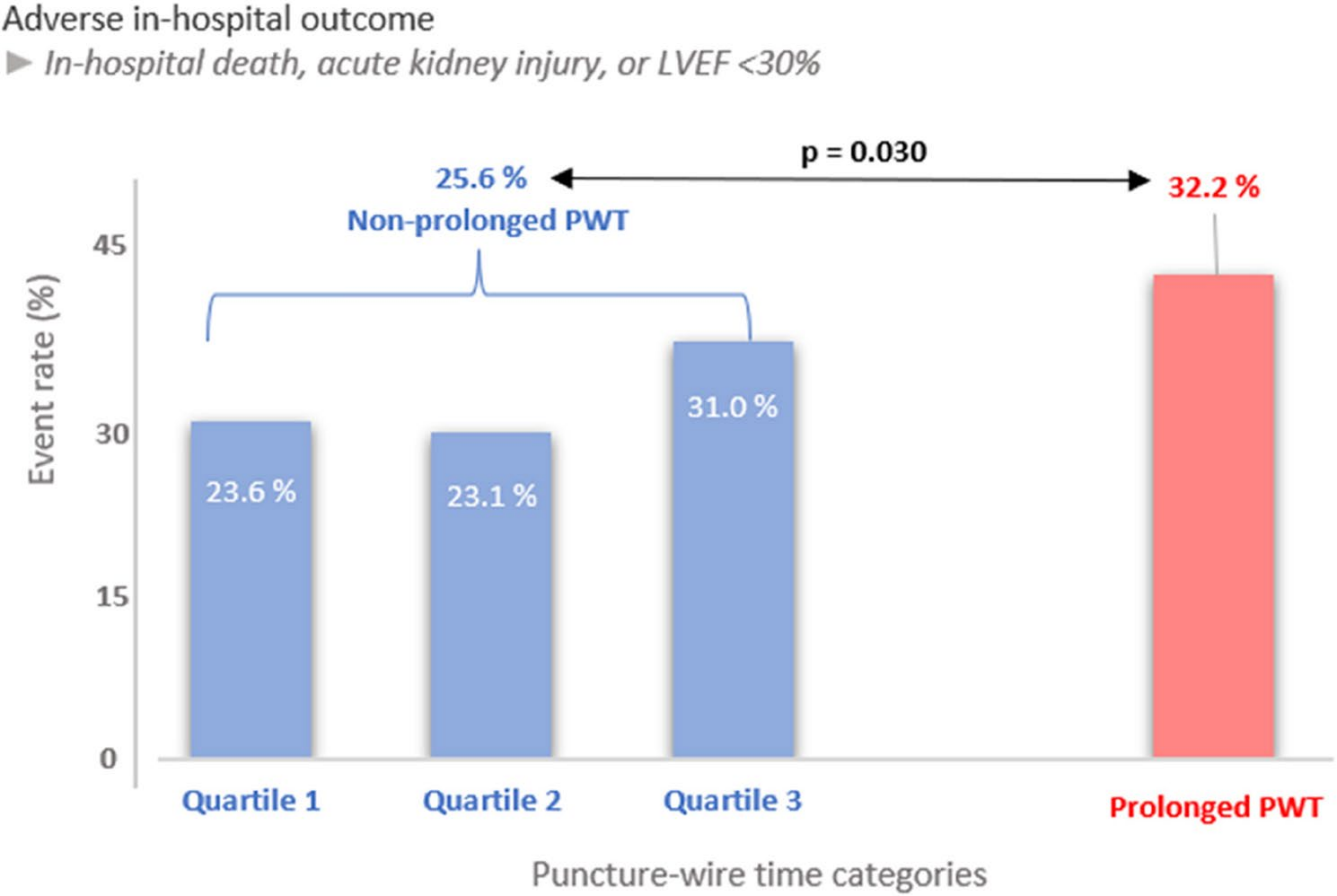
Comparison of adverse in-hospital outcome in patients with versus without prolonged puncture-wire time (PWT)

**Fig. 3:**
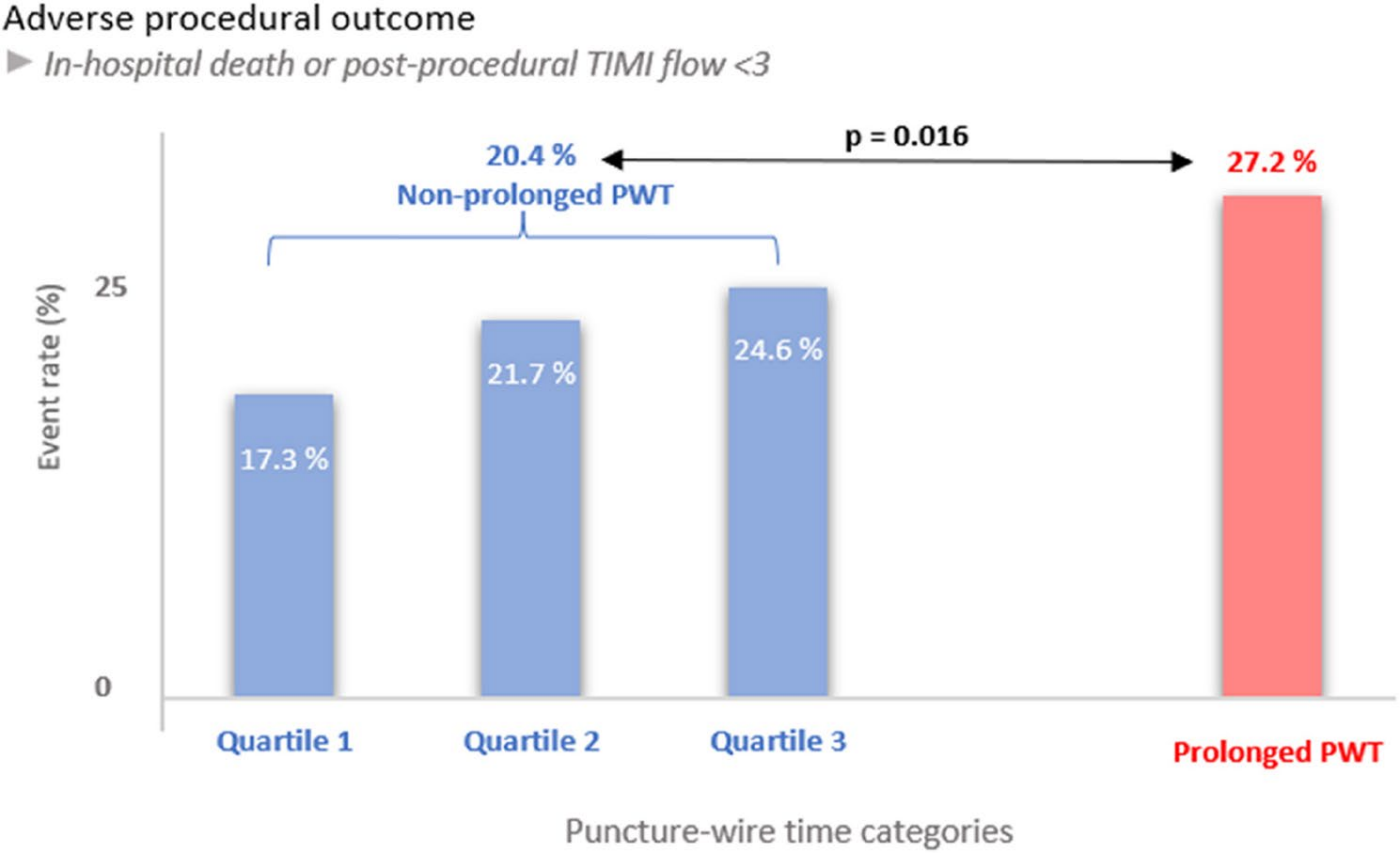
Comparison of adverse procedural outcome in patients with versus without prolonged puncture-wire time (PWT)

When assessing combined clinical endpoints, patients with prolonged PWT (quartile 4) showed significantly higher event rates (Table 3):

For the adverse in-hospital outcome, the incidence was 32.2% in patients with prolonged PWT versus 25.6% in those without prolonged PWT (OR 1.38, 95% CI 1.039–1.833, *p* = 0.030; Fig. 2), whereas in-hospital death, AKI, and LVEF < 30% did differ only numerically between the groups (Table 3). Similarly, for the procedural composite endpoint, the event rate was significantly higher in patients with prolonged PWT compared with patients without prolonged PWT (27.2% vs. 20.4%, OR 1.46, 95% CI 1.079–1.969, *p* = 0.016; Fig. 3). Among the individual components, post-procedural TIMI flow < 3 occurred significantly more often in patients with prolonged PWT (15.1% vs. 9.7%, OR 1.65, 95% CI 1.124–2.423, *p* = 0.014) (Table 3). Additional multivariable logistic regression analyses adjusting for age, arterial hypertension, diabetes mellitus, and previous CABG demonstrated that prolonged PWT was no longer independently associated with either the clinical composite endpoint (death, AKI, LVEF < 30%; adjusted OR 0.85, 95% CI 0.63–1.16, *p* = 0.316) or the procedural composite endpoint (death and post-procedural TIMI flow < 3; adjusted OR 0.82, 95% CI 0.59–1.14, *p* = 0.247). Maximum creatine kinase (CK max.) levels were significantly lower in patients with prolonged PWT (*p* = 0.005, Table 3).

**Table 3:** In-hospital clinical endpoints according to puncture-wire time

Endpoint	Non-Prolonged PWT (*n* = 937)	Prolonged PWT (*n* = 298)	OR (95% CI)	*p*-value
Composite endpoints				
In-hospital death, AKI, LVEF < 30% (n, %)	240 (25.6)	96 (32.2)	1.38 (1.039–1.833)	**0.030**
In-hospital death and post-procedural TIMI flow < 3(n, %)	191 (20.4)	81 (27.2)	1.46 (CI 1.079–1.969)	**0.016**
Individual endpoints				
In-hospital death (n, %)	124 (13.2)	52 (17.4)	1.390 (0.976–1.980)	0.071
AKI (n, %)	120 (12.8)	42 (14.1)	1.173 (0.802–1.717)	0.424
LVEF < 30% (n, %)	102 (10.9)	42 (14.1)	1.38 (0.937–2.033)	0.117
Post-procedural TIMI flow < 3(n, %)	91 (9.7)	45 (15.1)	1.65 (1.124–2.423)	**0.014**
CK max. (U/I)	2630.2 ± 3878.7	1949.2 ± 2314.0	-	**0.005**

*AKI *Acute Kidney Injury, *LVEF* Left ventricular ejection fraction, *TIMI* Thrombolysis in Myocardial Infarction flow, *CK* Creatinine Kinase, *CPR* Cardiopulmonary Resuscitation

Bold values indicate statistically significant differences (*p* < 0.05)

Receiver operating characteristic (ROC) analyses were performed to evaluate whether a clinically meaningful threshold for puncture-to-wire time could be identified for the two combined endpoints used in the present study. The discriminatory performance of puncture-to-wire time was modest, with AUC values of 0.564 (95% CI 0.528–0.600) for the composite endpoint of in-hospital death, acute kidney injury, or LVEF < 30%, and 0.567 (95% CI 0.529–0.605) for the composite endpoint of in-hospital death or post-procedural TIMI flow < 3 (Supplementary Figures S1 and S2). No robust clinically meaningful threshold could be identified. Therefore, the quartile-based definition of prolonged PWT was retained.

### Independent predictors for a longer puncture to wire time

In multivariable logistic regression analysis (Table 4), previous CABG (OR 2.75, 95% CI 1.24–6.092, *p* = 0.013) and the presence of cardiogenic shock at presentation (OR 1.7, 95% CI 1.11–2.714, *p* = 0.016) were identified as independent patient-related predictors of prolonged PWT.

**Table 4:** Multivariable logistic regression analysis for prolonged puncture -wire time

	Odds Ratio (95% CI)	*p*-value
Patient characterstics		
Age	0.999 (0.986–1.011)	0.822
Arterial hypertension	1.307 (0.929–1.838)	0.125
Diabetes mellitus	1.215 (0.859–1.719)	0.217
Hemoglobin level	0.968 (0.892–1.049)	0.425
Previous CABG	2.750 (1.242 − 6.092)	**0.013**
Clinical presentation		
Cardiogenic shock	1.734 (1.108–2.714)	**0.016**
CPR before or during angiography	0.960 (0.567–1.625)	0.878
CPR during angiography	1.135 (0.549–2.349)	0.732
Time intervalls and specification		
Procedure outside core working time	1.6327 (1.194–2.217)	**0.002**
FMC-to-door time	1.001 (1.000–1.003)	0.082
Door-to-puncture time	1.003 (1.001–1.005)	**0.007**
Procedural characteristics		
Primary access route radial	0.885 (0.615–1.272)	0.509
Arterial kinking / severe radial spasm	3.669 (1.986–6.778)	**< 0.001**
Change of primary access route from radial to femoral	2.539(1.381–4.665)	**0.003**
Challenging revascularization of culprit lesion	2.330 (1.319–4.116)	**0.004**
Direct intervention of culprit lesion	0.445 (0.201–0.988)	**0.047**

*CABG *Coronary Artery Bypass Grafting, *FMC* First medical contact, *CPR* cardiopulmonary resuscitation

Bold values indicate statistically significant differences (*p* < 0.05)

With regard to system-related factors, procedures performed outside core working hours were independently associated with prolonged PWT (OR 1.63, 95% CI 1194–2.217, *p* = 0.002), as was a longer door-to-puncture time (OR 1.003, 95% CI 1.001–1.005, *p* = 0.007), whereas first medical contact (FMC)-to-door time was not independently associated with prolonged PWT. 

**Fig. 4:**
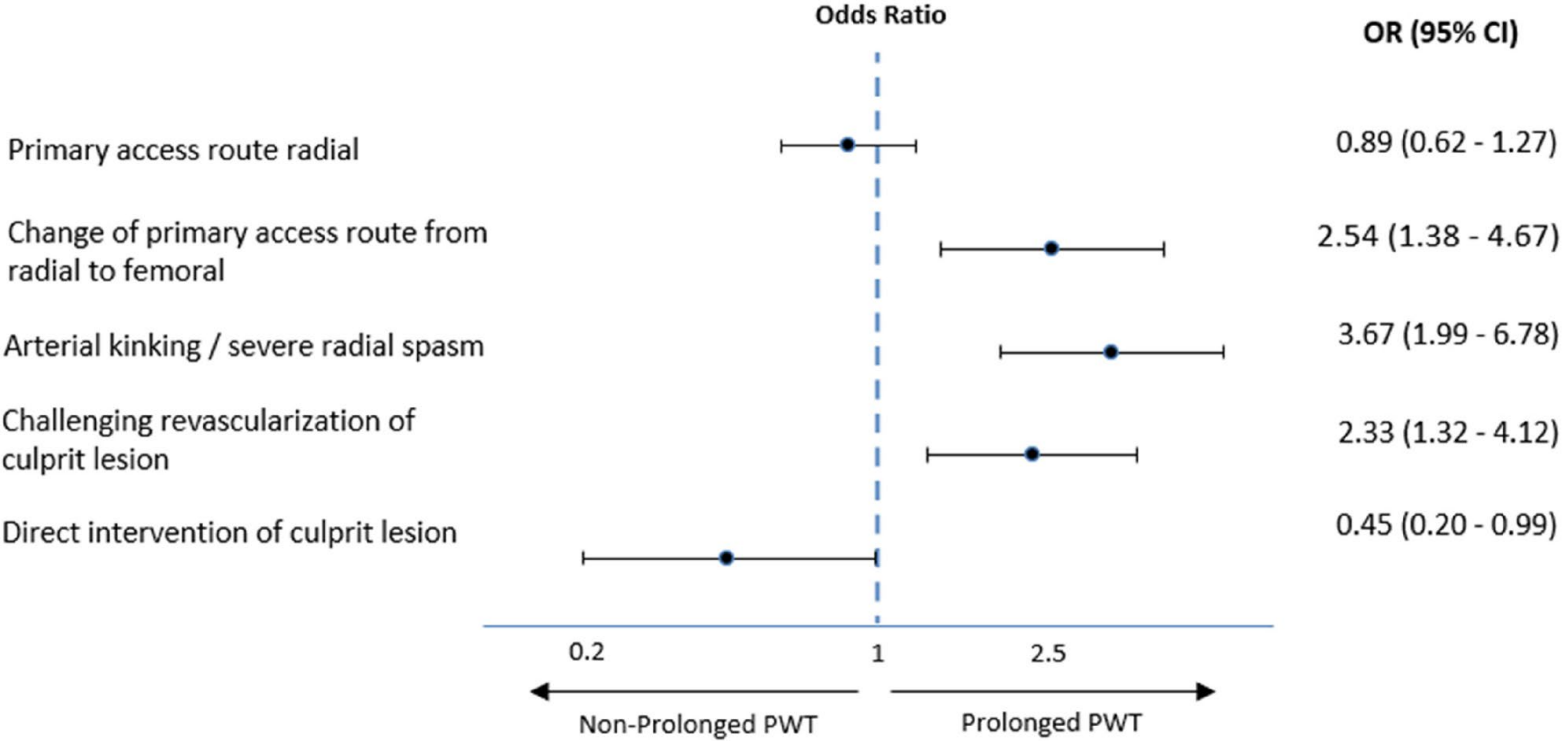
Procedural predictors of prolonged puncture-wire time. Forest plot showing adjusted odds ratios with 95% confidence intervals for procedural factors associated with prolonged puncture-wire time in multivariable logistic regression analysis. Odds ratios >1 indicate higher likelihood of prolonged PWT

Among procedural characteristics (Fig. 4), a change of access route from radial to femoral access independently predicted prolonged PWT (OR 2.54, 95% CI 1.381–4.665, *p* = 0.003). In addition, the occurrence of arterial kinking or severe radial spasm was strongly associated with prolonged wiring times (OR 3.67, 95% CI 1.986–6.778, *p* < 0.001). Finally, challenging revascularization of the culprit lesion was identified as an independent procedural predictor of prolonged PWT (OR 2.33, 95% CI 1.319–4.116, *p* = 0.004). In contrast, direct intervention of the culprit lesion was independently associated with a lower likelihood of prolonged PWT (OR 0.445, 95% CI 0.201–0.988, *p* = 0.045).

To further distinguish between factors present before the start of coronary angiography and those occurring during the procedure itself, two additional multivariable logistic regression analyses were performed (Tables 5 and 6). In the preprocedural model (Table 5), previous CABG (OR 2.677, 95% CI 1.276–5.619, *p* = 0.009), cardiogenic shock (OR 1.796, 95% CI 1.203–2.682, *p* = 0.004), presentation outside core working hours (OR 1.718, 95% CI 1.272–2.320, *p* < 0.001), and longer door-to-puncture time (OR 1.003, 95% CI 1.000–1.005, *p* = 0.016) emerged as independent predictors of prolonged PWT. In the procedural model (Table 6), radial-to-femoral crossover (OR 2.793, 95% CI 1.577–4.946, *p* < 0.001), severe radial spasm or arterial kinking (OR 3.535, 95% CI 2.000–6.250, *p* < 0.001), CPR during angiography (OR 1.748, 95% CI 1.041–2.934, *p* = 0.035), and challenging coronary access (OR 3.501, 95% CI 1.824–6.720, *p* < 0.001) were independently associated with prolonged PWT. In contrast, primary radial access (OR 0.661, 95% CI 0.494–0.886, *p* = 0.006) and direct culprit intervention (OR 0.327, 95% CI 0.160–0.669, *p* = 0.002) were associated with a lower likelihood of prolonged PWT.

**Table 5:** Preprocedural perdictors of prolonged puncture-wire time

	Odds Ratio (95% CI)	*p*-value
Patient characterstics		
Age	1.004 (0.992–1.016)	0.556
Arterial hypertension	1.356 (0.975–1.887)	0.071
Diabetes mellitus	1.171 (0.837–1.639)	0.358
Hemoglobin level	0.975 (0.902–1.055)	0.534
Previous CABG	2.677 (1.276–5.619)	**0.009**
Clinical presentation		
Cardiogenic shock	1.796 (1.203–2.682)	**0.004**
CPR before or during angiography	0.891 (0.542–1.465)	0.649
Time intervalls and specification		
Procedure outside core working time	1.718 (1.272–2.320)	**< 0.001**
FMC-to-door time	1.001(1.000–1.003)	0.062
Door-to-puncture time	1.003(1.000–1.005)	**0.016**

*CABG* Coronary Artery Bypass Grafting, *FMC* First medical contact, *CPR* cardiopulmonary resuscitation

Bold values indicate statistically significant differences (*p* < 0.05)

**Table 6:** Intraprocedural determinants of prolonged puncture-wire time

	Odds Ratio (95% CI)	*p*-value
Clinical presentation		
CPR during angiography	1.748 (1.041–2.934)	**0.035**
Procedural characteristics		
Primary access route radial	0.661 (0.494–0.886)	**0.006**
Arterial kinking / severe radial spasm	3.535 (2.000–6.250)	**< 0.001**
Change of primary access route from radial to femoral	2.793 (1.577–4.946)	**< 0.001**
Challenging revascularization of culprit lesion	3.501 (1.824–6.720)	**< 0.001**
Direct intervention of culprit lesion	0.327 (0.160–0.669)	**0.002**

*CPR* cardiopulmonary resuscitation

Bold values indicate statistically significant differences (*p* < 0.05)

## Discussion

In this real-world cohort of patients with STEMI undergoing primary PCI, prolonged PWT was associated with adverse in-hospital and procedural outcomes in unadjusted analysesThese associations were attenuated after adjustment for baseline characteristics, suggesting that prolonged PWT may partly reflect underlying patient risk and procedural complexity. Several patient-, system-, and procedure-related factors were independently associated with prolonged PWT, highlighting the multifactorial nature of intraprocedural delay.

In 1,235 consecutive STEMI patients undergoing primary PCI, we confirmed the hypothesis that prolonged PWT has a significant effect on short-term outcomes. Previous studies have consistently shown that prolonged wire crossing times are strongly associated with infarct size and mortality in STEMI, highlighting the relevance of intraprocedural delays [7–9]. While quality metrics have traditionally focused on door-to-balloon or FMC-to-device times, our findings emphasize that delays within the catheterization laboratory itself also contribute substantially to prognosis. Accordingly, our observation revealed that prolonged PWT was associated with impaired TIMI flow and higher rates of the adverse in-hospital composite outcome as well as the procedural composite outcome of in-hospital death and post-procedural TIMI flow < 3. However, several factors independently associated with prolonged PWT, including cardiogenic shock, prior CABG, and challenging lesion crossing, are themselves markers of increased procedural complexity and clinical severity. Nevertheless, one of the primary aims of the present study was to identify potentially modifiable procedural and system-related factors associated with prolonged PWT. While patient-related factors such as cardiogenic shock or prior CABG cannot be altered, procedural and organizational factors may offer opportunities to reduce treatment delays.

In our analysis, predictors and procedural determinants of prolonged PWT could be broadly categorized into patient-related factors (such as prior CABG and presence of cardiogenic shock), system-related factors (including presentation outside core working hours and prolonged pre- and in-hospital transfer intervals), and procedural factors.

Consistent with the previously described impact of CABG on door-to-balloon time [10], a similar effect was also observed when analyzing PWT alone. This likely reflects prolonged diagnostics and culprit lesion identification in patients with graft disease and complex native anatomy [11] Cardiogenic shock was another predictor, which is largely non-modifiable and likelyreflects clinical severity and the need for stabilization and laboratory preparation rather than deficiencies in care delivery.

System-level factors also contributed to prolonged PWT. Presentation outside core working hours was independently associated with prolonged PWT, suggesting persistent workflow limitations despite established STEMI networks. Prior reports similarly describe longer treatment intervals during off-hours [8, 9, 12] Large population-based data from Germany confirm improving in-hospital angiography times overall but persistent predictors of delay—including off-hours presentation, advanced age, and female sex—highlighting ongoing system challenges. While multidisciplinary strategies such as early cath lab activation and optimized transfer protocols reduce pre-puncture delays [13], they may have limited impact on intraprocedural phases. Our findings suggest that reduced staffing levels or limited experienced support during off-hours may impair efficiency once PCI has started. Importantly, PWT represents only one component of the overall STEMI treatment pathway, whereas substantial delays may already occur before arterial access is obtained, including during patient transfer, cath lab activation, and stabilization of critically ill patients.

The primary aim of our study was to identify procedure-related factors associated with prolonged PWT. Although the primary access route did not affect PWT, access-related complications—especially radial artery spasm or kinking—and radial-to-femoral crossover were strong predictors of delay. Consistent with previous reports describing radial-to-femoral crossover rates of approximately 8% in STEMI patients, crossover was required in 6% of our cohort [14–16]. In addition to our finding that crossover was independently associated with prolonged PWT, previous studies have also demonstrated an increase in access route-related complications and mortality [17]. However, this finding should be interpreted in the context of procedural complexity. Our registry did not allow differentiation between early crossover and crossover performed after prolonged unsuccessful radial attempts. Therefore, the observed association should not be interpreted as evidence against crossover itself. In selected patients, crossover may represent an appropriate strategy to facilitate successful reperfusion when radial access proves challenging.

Current guidelines recommend a primary radial access route in STEMI patients [18] due to the reduction of bleeding complications and consequently the reduction of mortality. In this context, more recent datasuggest that modern femoral access techniques, particularly when ultrasound-guided, may substantially mitigate bleeding risk, potentially narrowing the safety gap between access routes [19–21]. Importantly, primary radial access was not independently associated with prolonged PWT in our study. Rather, access-related complications such as severe radial spasm, arterial kinking, and access-site crossover were associated with procedural delay. Several factors predisposing to radial access failure- such as female gender, advanced age and vascular anatomy – are well described in large cohorts. However, many of these characteristics are also associated with an increased risk of femoral access-site complications [22–25]. Therefore, our findings should not be interpreted as supporting a routine femoral-first strategy but rather emphasize the importance of individualized access-site decision making. An individual risk assessment for radial access failure, for example using the Matrix score, may therefore be be useful when selecting the access site [22]. 

Importantly, direct intervention of the culprit lesion emerged as an independent predictor of shorter PWT, underscoring the importance of procedural prioritization. However, this finding should be interpreted cautiously, as reverse causation cannot be excluded. In some cases, direct intervention may have been possible because the culprit vessel was readily identifiable and lesion crossing was straightforward. Rapid reperfusion of the infarct-related artery remains the cornerstone of STEMI care [18]. In selected cases, operators may prioritize treatment of the culprit lesion before completion of a comprehensive assessment of the remaining coronary anatomy, thereby facilitating earlier reperfusion [26]. Accordingly, direct culprit intervention may reflect early angiographic recognition, prompt wiring, and ballooning, with comprehensive assessment deferred. Future studies may evaluate whether artificial intelligence-based approaches can improve the identification of patients at risk for prolonged procedural delays and support individualized procedural planning.

### Limitations

Our study is limited by its retrospective, single-center design and the potential for residual confounding. Timing metrics, such as puncture and wire crossing, were derived from procedural documentation. Despite standardized definitions, these metrics are subject to measurement error and inter-operator variation. Time intervals prior to angiography showed substantial variability with marked skewness and outliers. This was largely attributable to the inclusion of patients with out-of-hospital cardiac arrest, some of whom were admitted without return of spontaneous circulation and therefore experienced prolonged pre-hospital and in-hospital stabilization phases. These extreme cases resulted in wide standard deviations and should be interpreted in the context of the overall clinical heterogeneity of a real-world STEMI cohort. Although the magnitude of this variability was greater than anticipated, we believe that it reflects the broad spectrum of clinical presentations encountered in contemporary STEMI care, particularly with regard to pre-hospital and early in-hospital treatment intervals. In addition, data specifically addressing puncture-wire time remain scarce and, to our knowledge, no established threshold for clinically relevant PWT prolongation currently exists. Therefore, the definition of prolonged PWT based on the highest quartile was selected pragmatically to identify patients with the longest procedural delays within our cohort. The identified cut-off is cohort-specific and may not be directly transferable to other institutions or healthcare systems. Another limitation is that the timing of crossover decisions was not systematically documented. Consequently, we were unable to distinguish between early crossover and delayed crossover after prolonged unsuccessful radial attempts. In addition, prolonged PWT may partly represent a marker of procedural complexity and patient severity rather than a directly modifiable cause of adverse outcomes. Furthermore, although we report associations between prolonged PWT and adverse in-hospital events, causality cannot be inferred from observational data and some of the clinical endpoints may be driven by baseline severity (e.g., cardiogenic shock) rather than the delay itself. Some variables identified in our analysis, such as radial-to-femoral crossover, arterial spasm, and challenging lesion crossing, occur during the puncture-to-wire interval itself and should therefore be interpreted as procedural determinants rather than purely preprocedural predictors.

## Conclusion

In summary, prolonged PWT in STEMI is driven by a combination of anatomical/procedural (kinking/spasm, crossover, challenging coronary anatomy), patient factors (prior CABG, cardiogenic shock) and system factors (off-hours staffing, pre-puncture delays). While many patient-level risks are non-modifiable, anticipating difficult radial access and making an early decision about the access route, together with targeted system-level process improvements, represent realistic strategies to shorten PWT and thereby potentially improve reperfusion quality. Prospective evaluation in randomized or pragmatic trials is needed to determine whether tailored access strategies and workflow interventions will lead to better procedural success and clinical outcomes.

## Supplementary Information

Supplementary material 1.

## Data Availability

The datasets used and/or analyzed during the current study are available from thecorresponding author on reasonable request.
