# Supplementary material for: Predictors of a prolonged puncture-wire time in patients with ST-Elevation Myocardial Infarction (STEMI)
Source: BMC Cardiovasc Disord. 2026 Jul 7;26:574. doi: 10.1186/s12872-026-06220-x (PMC13343796; doi:10.1186/s12872-026-06220-x)

**Supplementary Figure S1**

Receiver operating characteristic (ROC) curve of puncture-to-wire time for prediction of the clinical composite endpoint. The area under the curve (AUC) was 0.564.

**Supplementary Figure S2**

Receiver operating characteristic (ROC) curve of puncture-to-wire time for prediction of the procedural composite endpoint. The area under the curve (AUC) was 0.567.

**Supplementary Figure 1**


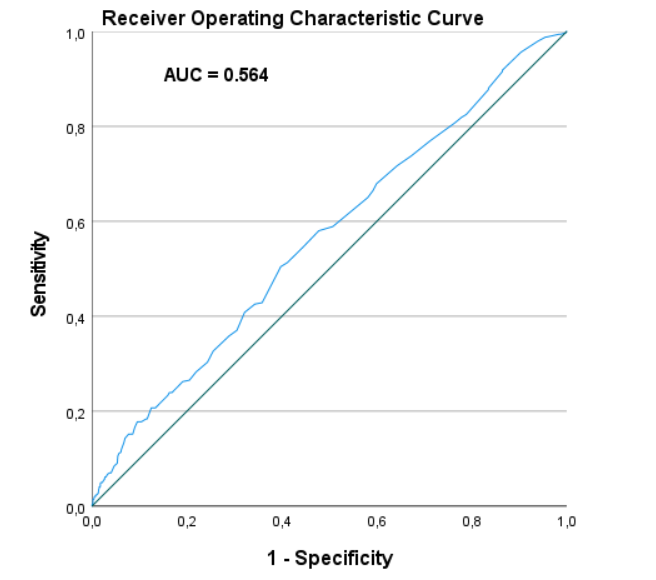


**Supplementary Figure 2**


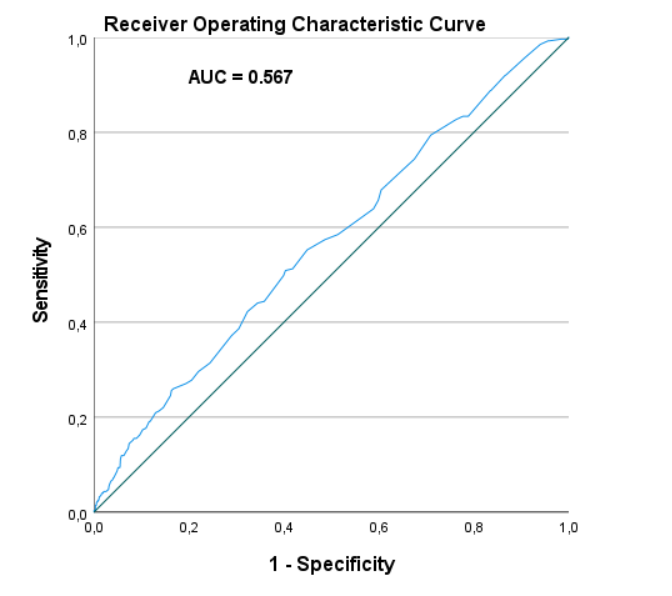

Supplement: Supplementary file 1 — Supplementary material 1. [file 12872_2026_6220_MOESM1_ESM.docx]
